# Biotin Supplementation—The Cause of Hypersensitivity and Significant Interference in Allergy Diagnostics

**DOI:** 10.3390/nu17152423

**Published:** 2025-07-24

**Authors:** Kinga Lis

**Affiliations:** Department of Allergology, Clinical Immunology and Internal Medicine, Ludwik Rydygier Collegium Medicum in Bydgoszcz, Nicolaus Copernicus University in Torun, ul. Ujejskiego 75, 85-168 Bydgoszcz, Poland; kinga.lis@cm.umk.pl

**Keywords:** biotin, allergy, hypersensitivity, immunochemical methods, interferences

## Abstract

Biotin (vitamin B7) is a common, naturally occurring water-soluble vitamin. It belongs to the broad group of B vitamins. It is a common ingredient in dietary supplements, cosmetics, medicines, and parapharmaceutical preparations administered orally or applied topically (to the skin, hair, nails). The problem of the relationship between vitamin B supplementation and sensitivity seems to be multi-threaded. There is little literature data that would confirm that oral vitamin B supplementation or local exposure to biotin is a significant sensitizing factor. Moreover, it seems that allergy to vitamin B7 is very rare. It is possible, however, that the relationship between biotin and hypersensitivity is not limited to its direct action, but results from its essential metabolic function. Vitamin B7, as a cofactor of five carboxylases, affects the main pathways of cellular metabolism. Both deficiency and excess of biotin can result in metabolic disorders, which can have a significant impact on the homeostasis of the entire organism, including the efficient functioning of the immune system. Dysregulation of immune systems leads to its dysfunctional functioning, which can also lead to sensitization to various environmental antigens (allergens). Biotin is also used as an element of some methodological models in immunochemical tests (in vitro diagnostics), including methods used to measure the concentration of immunoglobulin E (IgE), both total (tIgE) and allergen-specific (sIgE). For this reason, vitamin B7 supplementation can be a significant interfering factor in some immunochemical tests, which can lead to false laboratory test results, both false positive and false negative, depending on the test format. This situation can have a direct impact on the quality and effectiveness of diagnostics in various clinical situations, including allergy diagnostics. This review focuses on the role of biotin in allergic reactions, both as a causative factor (allergen/hapten), a factor predisposing to the development of sensitization to various allergens, and an interfering factor in immunochemical methods used in laboratory diagnosis of hypersensitivity reactions and how it can be prevented.

## 1. Introduction

Vitamins are a group of organic chemical compounds of various structures that are essential for the proper functioning of a living organism. The term “vitamin” comes from the Latin word “*vita*” (life) and the name “*amine*”—a characteristic functional group found in the first described vitamin (thiamine, vitamin B1). The name “vitamin” was proposed in 1913 by Polish biochemist Kazimierz Funk after he discovered and described the first recognized vitamin (B1) [1].

In terms of physical properties, vitamins are divided into water-soluble (vitamin B group and vitamin C) and fat-soluble (vitamins A, D, E, K). Biotin is one of the B vitamins (vitamin B7) [2].

Biotin, like other vitamins, can come from natural sources or can be obtained by chemical synthesis. Vitamins as ingredients of supplements, medicines or cosmetics can be taken orally/enterally, by injection (subcutaneous, intramuscular, intradermal, intravenous) or applied topically [3]. Vitamin B7, apart from therapeutic applications, is a common ingredient in cosmetics and supplements from the “beauty” area, which are used even in very high doses to improve the condition of the skin, hair and nails. Exposure to this vitamin is common [4].

Although vitamin allergies are considered rather rare, the commonness and chronicity of their supplementation and exposure to high doses may be a potential risk factor for sensitization. Biotin, like other water-soluble vitamins, is easily absorbed from the intestines, from where it is transported to tissues [5,6]. In addition, vitamin B7 is readily bound to various proteins, whereby as a potential hapten, it theoretically could acquire the properties of a complete antigen. The biotin–avidin complex has been proven to be immunogenic [7,8]. It seems likely, therefore, that biotin may pose a potential risk of sensitization. It is possible that it is a little-known hidden allergen, which is worth considering in a situation where the patient manifests symptoms of hypersensitivity and the causative factor remains difficult to identify. The ability of biotin to bind to various proteins, including avidin and steptavidin, is also used in immunochemical techniques used, among others, in in vitro diagnostics of allergies and hypersensitivity. These techniques are sensitive to the concentration of vitamin B7 in the biological material being tested. High concentrations of biotin can interfere in these studies by blocking or enhancing immunochemical reactions. This can significantly hinder or prevent allergy diagnostics [9,10].

This review focuses on the role of biotin in allergic reactions, both as a causative factor (allergen/hapten), a factor predisposing to the development of sensitization to various allergens, and an interfering factor in immunochemical methods used in laboratory diagnosis of hypersensitivity reactions and how they can be prevented.

## 2. Biotin

Biotin, like most of the B vitamins, was discovered during studies on factors influencing yeast colonies. In 1935, Fritz Kögl and Benno Tönnis [11] isolated biotin from duck egg yolk, identifying this substance as one of the components that, when added in small amounts to the medium, stimulates yeast growth.

This vitamin is known not only as biotin, but also as vitamin H, vitamin B7 and vitamin B11. This is due to the fact that this substance was discovered by different independent teams and only the precise knowledge of the structure of the biotin molecule showed that it is the same substance [12,13]. The first, historical name of biotin, “vitamin H”, comes from the German words “*Haut*” and “*Haar*” and is directly related to the originally observed symptoms, such as poor condition of the skin (German “*Haut*”) and hair (German “*Haar*”) or fur, which were associated with a deficiency of this nutrient in the diet of animals and humans [13,14]. Vitamin H was the name given by Paul Gyorgy to the substance he discovered during his research conducted in 1933–1939 [15,16]. This vitamin also functioned simultaneously as coenzyme R [17]. The name “biotin” comes from the Greek word “bios” (“living”) and the general chemical suffix used in organic chemistry “-in” and was introduced in the 1940s as a common name for substances studied by different, independent research teams, after it was established that each of them was conducting research on the same substance isolated from different sources [17,18,19,20].

**Figure 1:**
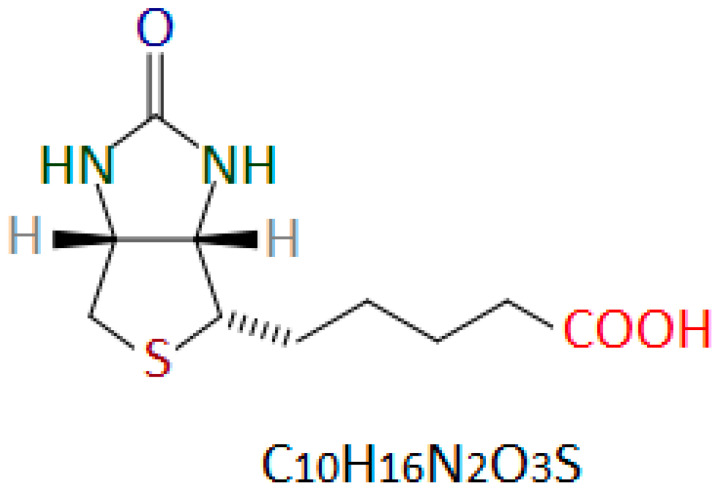
Chemical structure of D-(+)-biotin. Author’s own drawing based on [22,23,24].

The structure of biotin was determined in 1942 by Vincent du Vigneaud [21]. Biotin is a heterocyclic organic chemical compound from the B vitamin group. The vitamin B7 molecule contains a system of condensed imidazolidine and thiolate rings with an alkyl chain terminated with a carboxyl group (valeric acid chain) (Figure 1) [14,22].

Vitamin B7 is a water-soluble substance, insoluble in organic solvents, stable at pH 4 to 9. Biotin is stable at high temperatures (the melting point of biotin is 229–232 °C) and does not decompose under the influence of sunlight. At room temperature, it is a solid, odorless substance. In these conditions, it has the form of a white crystalline powder or colorless crystals [13,22,25].

Biotin naturally occurs in various isomeric forms. Only the D-(+)-biotin isomer (Figure 1) is biologically active [26,27]. D-(+)-biotin is the only form permitted for use in dietary supplements and as a food additive [28,29,30].

The first reports of indigestion of raw egg white date back to the end of the 19th century [31]. However, it was only intensive research on this issue, initiated in the second decade of the 20th century, that made it possible to find the cause and solution to this problem. In 1916, Bateman [32] observed that dogs, cats, rabbits and humans fed a diet rich in raw egg whites, which were the main source of protein, experienced increased hair loss and various skin lesions. Similar observations were made in 1927 by Margarete Boas [33]. She noted that rats fed large amounts of raw egg as the sole source of protein developed dermatitis (skin rashes), baldness and loss of muscle coordination. Raw egg white was identified as a consistent factor for both of these observations. On this basis, it was concluded that raw egg white probably contains some factor that has a toxic effect and causes a deficiency of some important dietary factors. This deficiency results in damage to the skin, hair or fur and the nervous system. In extreme cases, it can lead to the death of the animal. This then unknown, important nutrient, the deficiency of which leads to the dysfunctions described earlier, was named “vitamin H” [12,13].

The mechanism of the toxic effect of raw egg white on the organism of the animal fed with it was explained by Paul György’s team [34] in the early 1940s. These researchers showed that the cause of vitamin B7 deficiency in animals whose diet is rich in raw egg white is caused by the binding of biotin by avidin present in raw egg white. Avidin is a glycoprotein found in egg whites, which very specifically and tightly binds biotin. This property prevents the absorption of biotin from raw eggs but has no effect on the availability of this vitamin from heat-treated eggs, because heating denatures avidin, which causes it to lose its ability to bind biotin [35].

Vitamin B7 is synthesized by plants and some bacteria and yeasts [22]. Animals, including humans, do not produce biotin. Although vitamin B7 is produced endogenously by bacteria in the large intestine, the basic requirement for this vitamin is met by the supply from food [6,22]. Biotin from the diet is absorbed in the small intestine, while biotin synthesized by the gut microbiome is absorbed in the large intestine [5,6,22,36]. Biotin absorption from the intestine occurs through a carrier-dependent process that involves the sodium-dependent multivitamin transporter (SMVT), a membrane protein responsible for transporting vitamins into the body’s cells. The SMVT transports biotin (vitamin B7), pantothenic acid (vitamin B5), and lipoic acid [5,6,22,36]. Since vitamin B7 is bound in animal tissues, the dietary source of this vitamin can be food products of both plant and animal origin (Table 1). Biotin naturally present in food can be available in a free state (e.g., in milk and vegetables) or bound to protein (e.g., in meat and yeast). Biotin in a free state is more easily absorbed from the gastrointestinal tract, which facilitates its assimilation. The common occurrence of biotin in food products means that vitamin B7 can be obtained through diet in the amount that is necessary to maintain human cell homeostasis [13,36,37].

A well-balanced diet provides 30–70 µg of biotin per day. The recommended daily intake of biotin is 30–70 µg for an adult and 5 µg for a newborn. This means that biotin deficiency in healthy people who eat properly does not occur in principle. Biotin deficiency may, however, occur in states of increased demand for vitamin B7 (e.g., pregnancy, long-term parenteral nutrition), in some hereditary metabolic diseases, in states of malnutrition, an undesirable effect of therapy with anticonvulsants or oral steroids, in patients with Crohn’s disease, in alcoholics and in people who consume large amounts of raw eggs. Symptoms of vitamin B7 deficiency may result in general metabolic disorders, muscle pain, fatigue and damage to the structure of hair, nails and/or skin [39,40,41].

## 3. Hypersensitivity to Biotin

### 3.1. Clinical Reports of Biotin Hypersensitivity

According to available analyses, biotin contained in cosmetics, drugs, supplements or fortified foods does not seem to cause hypersensitivity reactions of any type. No reports of such events have been published so far [42].

In the published literature, only two cases of hypersensitivity reactions to biotin or precursor substances used for the synthesis of vitamin B7 can be found. In both reported cases, the reaction was not related to vitamin B7 supplementation or the use of cosmetics or foods fortified with this vitamin but developed as a result of occupational exposure to biotin synthesis intermediates or vitamin B7 [43,44].

#### 3.1.1. Case 1—Occupational Contact Allergy to Biotin [43]

In 1942, Keller [43] described a case of contact allergy, the symptoms of which developed in a pharmaceutical worker as a result of occupational exposure to components of a B vitamin complex. The patient had recurrent red, itchy spots on his arms and hands. The lesions always appeared during the performance of professional duties. The man underwent skin patch tests with each substance to which the patient was exposed during work, including the full B vitamin complex and each vitamin from this mixture separately.

A positive result was observed for the full vitamin B complex and for biotin and nicotinic acid. The skin reaction to the vitamin B complex and biotin was much stronger and lasted much longer than to nicotinic acid. The escalation of changes was particularly significant at the biotin application site, where a diffuse erythema of a significant surface area, swelling and a centrally located weal occurred. The site of testing with biotin was painful. The patient still felt pain for 36 h after the changes had subsided. In comparison, nicotinic acid caused only erythema, without swelling, and these changes subsided within an hour [43].

Based on the clinical history and the results of additional tests, Keller [43] concluded that the main cause of the skin changes occurring in the patient was contact hypersensitivity to biotin caused by occupational exposure to this vitamin.

#### 3.1.2. Case 2—Occupational Contact Allergy to Biotin Substrate [44]

In 1998, Nishioka et al. [44] described a case of hypersensitivity reaction to a biotin-producing substrate that developed as a result of occupational exposure in a 45-year-old man. The patient complained of itchy, reddish eruptions on his face and hands that had been present for a year. For about 18 months, the man had worked in a factory that manufactured biotin precursor substances.

The patient’s peripheral blood count showed mild eosinophilia. The total immunoglobulin E (tIgE) concentration was very high (1000 kU/L). Specific IgE antibodies (sIgE) were found for cedar pollen allergens, house dust mite allergens and Candida fungi spore allergens [44].

**Figure 2:**
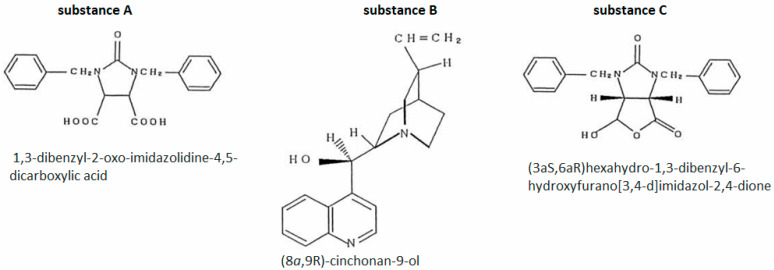
Case 2—substances used in epidermal patch tests [44].

The man underwent epidermal patch testing with three substances (Figure 2) to which the patient had been exposed in connection with his work. All of these substances were used in the factory to produce biotin precursors [44].

Positive results were obtained for substance B and substance C. The reactions for substance C were much stronger, occurred at lower concentrations and were repeatable in the second testing cycle [44].

Based on the patient’s clinical history and the results of additional tests, Nishioka et al. [44] concluded that the cause of the skin lesions in the man was contact, occupational exposure to substances used to synthesize the biotin precursor. Additionally, the validity of this diagnosis seems to be confirmed by the fact that the skin lesions completely disappeared after the patient changed jobs to one in which he was not exposed to these substances.

**Figure 3:**
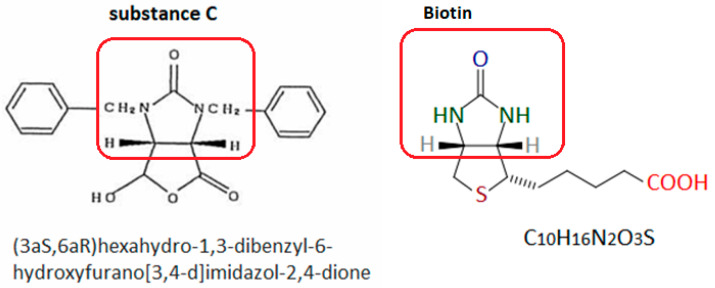
Substance C [44] and biotin. The red frame indicates the group probably responsible for inducing the hypersensitivity reaction. Author’s own figure based on [24,44].

Nishioka et al. [44] also suggested that a cyclic fragment of the substance C molecule is responsible for causing the hypersensitivity reaction (Figure 3). Since this type of grouping is also present in the vitamin B7 molecule, it seems likely that it may also be the cause of biotin hypersensitivity. Unfortunately, these authors did not perform skin tests with biotin on the patient. This makes it impossible to determine whether the patient was also allergic to vitamin B7.

### 3.2. Other Aspects of Biotin Hypersensitivity

Biotin is a cofactor of carboxylase enzymes: pyruvate carboxylase (EC 6.4.1.1), acetyl-CoA carboxylase 1 and 2 (1 and 2, EC 6.4.1.2), propionyl-CoA carboxylase (EC 6.4.1.3) and methylcrotonoyl-CoA carboxylase (EC 6.4.1.4). These enzymes play an important regulatory role at the cellular level in various metabolic pathways dependent on carboxylases (e.g., gluconeogenesis, fatty acid synthesis, amino acid metabolism) [36,45,46,47]. The multienzymatic metabolic cycle in which biotin participates is called the “biotin cycle”. Biotin in the biotin cycle is repeatedly used in the activation processes of subsequent carboxylases [36,45,46,47]. The enzymes holocarboxylase synthetase (HCS) (EC 6.3.4.10) and biotinidase (BT) (EC 3.5.1.12) play a key role in biotin metabolism [36,45,46,47,48]. HCS catalyzes the covalent binding of biotin to specific lysine residues of carboxylases, while BT releases biotin from biotinylated peptides during carboxylase turnover. Biotin released from old carboxylases is then used to biotinylate new carboxylases. Due to the closed nature of the process, a defect in the biotin cycle results in multiple deficiencies of carboxylase enzymes. Due to the metabolic importance and interconnectedness of these enzymatic processes, biotin deficiency is potentially fatal [36,45,46,47,48]. Biotin status in the body also significantly affects the homeostasis of the immune system by regulating the balance of inflammatory and anti-inflammatory processes, depending on and independent of biotin-dependent carboxylases [36]. It regulates the synthesis of important transcription factors (e.g., nuclear factor kappa-light-chain-enhancer of activated B cells (NF-κB), Specificity protein 1/3 (Sp1/3)) [22,45,49] and cytokines (e.g., interferon gamma (IFN-γ), tumor necrosis factor alpha (TNF-α), interleukin (IL) 17 (IL-17), IL-12p40, IL-23, IL-4, IL-2, IL-1β) [45,50,51,52,53]. It seems very likely that metabolic abnormalities associated with dysregulation of biotin levels in the body may play an important role in dysregulation of immune pathways. This may be involved in the mechanisms of development of hypersensitivity to various allergens and/or haptens and modulation of these reactions. Based on the available data discussed below [54,55], it seems that both deficiency and excess of vitamin B7 may be the cause of these dysfunctions. As the available data show, the relationship between biotin and hypersensitivity reactions should probably be considered in a much broader context than direct hypersensitivity to vitamin B7 and/or its precursors or derivatives.

#### 3.2.1. Biotin Deficiency and Hypersensitivity Reactions

Biotin deficiency causes, among other things, adverse changes in the condition of the skin. Skin damage may predispose to the development of contact hypersensitivity to various allergens or haptens. Kuroishi et al. [54] demonstrated in an experimental mouse model that animals fed a diet low in biotin more often develop contact hypersensitivity to nickel compared to animals on a diet with a balanced content of vitamin B7.

The study cited above [54] of the effect of biotin status on nickel (Ni) allergy was performed in two ways: in vivo and in vitro.

In the in vivo branch, mice were fed a basal diet (control-CD) or a biotin-deficient diet (BD) for 8 weeks and were sensitized intraperitoneally with NiCl_2_ and lipopolysaccharide injection. Ten days after sensitization, NiCl_2_ was injected intradermally into the auricle and ear swelling was measured at the injection site. It was noted that ear swelling after NiCl_2_ injection was significantly greater in BD mice than in CD mice and persisted significantly longer in them [54].

Adaptive transfer of splenocytes from sensitized BD mice to CD mice (passive sensitization) was also performed. Intradermal NiCl_2_ tests (as before) were performed 24 h after transfer. It was observed that the transfer of splenocytes from BD mice to CD mice induced nickel allergy in control mice. Interestingly, the ear swelling after NiCl_2_ injection was always significantly greater in donor mice (BD) than in recipient mice (CD) [54].

All mice were then supplemented with biotin. It was observed that this effect reduced the ear swelling induced by NiCl_2_ injection in both BD and CD mice. The effect was dose-dependent [54].

In the in vitro branch, a four-week cell culture of the J774.1 mouse macrophage line was used in conditions of sufficient amounts of biotin (control-CM) or in conditions of biotin deficiency (BDM). Differences were assessed by analyzing the production of interleukin 1 beta (IL-1β) and the expression of IL-1β mRNA by the cultured cells. It was found that IL-1β production and IL-1β mRNA expression were significantly higher in J774.1 BDM cells than in J774.1 CM cells. Moreover, biotin supplementation of BDM cell cultures (in vitro) inhibited the increase in IL-1β production [54].

In another track of the in vitro branch, IL-1β secretion by splenocytes collected from both groups of mice (CD and BD) was assessed. Splenocytes from BD mice produced significantly more IL-1β than splenocytes from CD mice [54].

Kuroishi et al. [54] concluded that the obtained results indicate that biotin deficiency predisposes mice to the development of nickel allergy by the mechanism of increased IL-1β production. This suggests that vitamin B7 supplementation may have a therapeutic and preventive effect in metal allergies.

#### 3.2.2. Biotin Overdose and Hypersensitivity Reactions

Sakurai-Yageta et al. [55] investigated the association between blood biotin levels and IgE-mediated allergic sensitization to aeroallergens in a population of 411 Japanese schoolchildren aged 6 to 12 years. The children were selected from a group of 843 schoolchildren using a questionnaire based on the International Study of Asthma and Allergies in Childhood (ISAAC) [56]. This questionnaire assesses the occurrence of allergic diseases and environmental and lifestyle factors that may influence the development of allergies or be related to its occurrence. The serum of school children included in the study was measured for biotin, total immunoglobulin E (tIgE), and specific IgE (sIgE) for common airborne allergens (*Dermatophagoides pteronyssinus*, black mold, cat dander, dog dander, hamster dander, cedar pollen, orchard grass) and food allergens (egg white). These researchers observed a weak positive correlation between biotin concentration and total IgE and specific IgE for cedar pollen, cat allergens, and egg white allergens. This relationship was particularly evident in children allergic to cedar pollen. In addition, the mean serum biotin concentration in children with cedar pollinosis was significantly higher than in children with other forms of cedar pollen allergy or sensitized to other allergens.

Based on these observations, Sakurai-Yageta et al. [55] suggested that the association of high serum biotin concentration with sensitization to airborne allergens (especially cedar pollinosis) or egg protein is probable. Unfortunately, the mechanism of this phenomenon remains unexplained.

#### 3.2.3. Intestinal Microbiota as an Endogenous Source of Biotin in Relation to Allergy and Asthma

The intestinal microbiota is a valuable, endogenous source of vitamin B7 [6,22,57]. As mentioned earlier, the absorption of biotin produced by the colonic microbiota occurs at the site of its production [6,36]. There is no consistent data on the extent to which endogenous biotin synthesis by intestinal bacteria satisfies the daily human requirement for this vitamin [5,6,22]. The composition of the intestinal microbiota directly impacts the amount of endogenously produced vitamin B7 [6,22].

The link between changes in the composition of the intestinal microbiota and disruptions in immune system homeostasis has been the subject of intensive research in recent years [22,57,58,59,60,61]. It has been noted that intestinal dysbiosis may be associated with various diseases and other pathological psychosomatic conditions, such as inflammatory bowel disease [57,62,63], irritable bowel syndrome [57,58,59,64,65], obesity [57,58,59,66,67], type 2 diabetes [57,58,59,68,69], depression [57,58,59,68,70,71], insomnia [68,72], and various immune disorders and deficiencies [57,68,73,74,75], including autoimmune disorders [68,74,75], atopic dermatitis [76], allergy [77,78,79,80,81,82,83,84], or asthma [84,85,86,87,88,89].

However, the etiopathogenesis of these disorders in connection with the intestinal microflora and the metabolites it secretes has not yet been clearly determined. It cannot be ruled out that abnormal metabolism of the intestinal microflora, resulting from dysbiosis, causes disturbances in the secretion of biotin, an important cofactor of numerous enzymes involved in a number of key metabolic processes, and may be important in maintaining the balance of immunological processes [22,90]. However, there is no sufficient data to confirm or exclude the existence of such a relationship.

#### 3.2.4. Biotin in the Context of Nutraceutical Strategies to Support the Treatment of Asthma and Other Allergic Diseases

Biotin is considered a likely important component of nutraceutical programs supporting the treatment of asthma [91]. According to McCarty et al. [91], biotin in high doses can mimic and potentially enhance the activating effect of nitric oxide (NO) on soluble guanylate cyclase (sGC). NO is a ligand for sGC, an intracellular enzyme that catalyzes the conversion of guanosine triphosphate (GTP) to cyclic GMP (cGMP). Through activation of protein kinase G, cGMP reduces smooth muscle tone in the bronchi or blood vessels. Thanks to these activities, NO participates in many signaling processes in both physiological and pathological conditions [92]. Available data indicate that sGC expression and activity are reduced in individuals with asthma, which likely results in airway hyperresponsiveness [91,93]. According to McCarty et al. [92], biotin at concentrations approximately twice the physiological level acts as an activator of sGC, increasing cGMP production by approximately 2–3 times. It therefore seems likely that biotin supplementation potentially relaxes bronchial smooth muscle, which may dilate the airways, reduce dyspnea, and alleviate the increased reactivity of the bronchial smooth muscle characteristic of asthma [91,92].

McCarty et al. [92] point out, however, that it is uncertain whether biotin also enhances the sGC response with simultaneous exposure to NO. This is important because in people with asthma, NO concentrations in the airways are high, which results from the chronic inflammation characteristic of this disease [94,95,96,97].

The idea of using biotin in nutraceutical strategies to support asthma treatment is relatively new, and although it appears that biotin in high doses may have practical therapeutic potential in diseases associated with chronic inflammation and bronchial hyperresponsiveness, the limited number or even absence of studies in this area, including animal models [92], significantly complicates conclusions. This is especially true considering that, as shown by Kuroishi et al. [54] and Sakurai-Yageta et al. [55], both biotin deficiency and excess may be unfavorable in terms of predisposition to the development of allergies, including allergies to airborne allergens.

## 4. Biotin Supplementation—A Factor Complicating In Vitro Allergy Diagnosis

The previously mentioned interaction of biotin with avidin, which prevents the absorption of vitamin B7 from raw eggs and is unfavorable or even toxic from a dietary point of view, has found significant application in some techniques used for the isolation and visualization of various antigens, for drug delivery, lymphocyte stimulation and various immunochemical and histochemical diagnostic methods [98,99,100,101].

**Figure 4:**
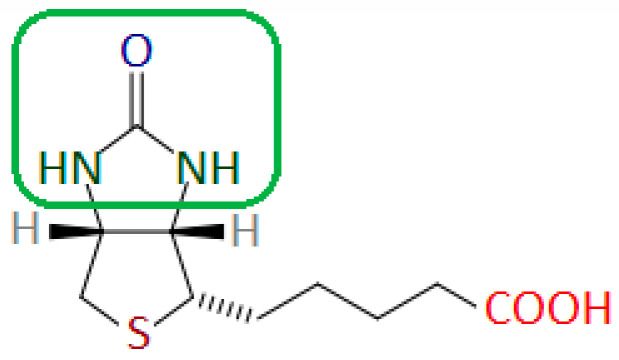
Biotin molecule with the fragment responsible for binding to avidin and streptavidin marked in the green frame. Author’s own drawing based on [22].

Biotin has a strong affinity for avidin and streptavidin—a tetrameric protein produced by *Streptomyces avidinii* bacteria. The imidazolidine fragment of the biotin molecule is responsible for binding to streptavidin or avidin (Figure 4) [22].

**Figure 5:**
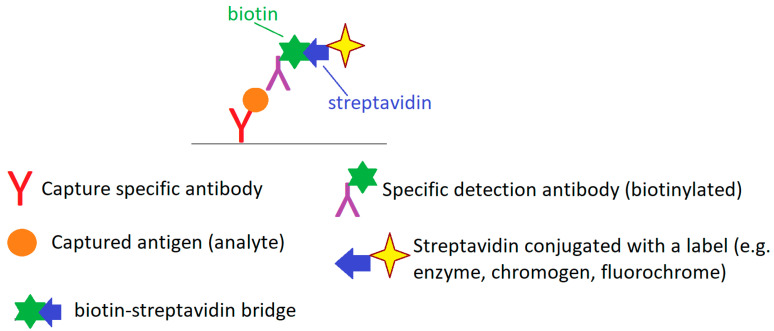
A biotin-streptavidin ELISA format—basic scheme. Author’s own drawing based on [102,103].

The scheme of the enzyme-linked immunosorbent assay (ELISA) using biotin and streptavidin (or less frequently avidin) (Figure 5) uses the strong affinity of biotin and streptavidin (avidin) and the possibility of conjugating both biotin and streptavidin with various substances, including dyes, fluorochromes, enzymes and various immunocompetent molecules [102].

The use of a biotin–avidin or biotin–streptavidin bridge in immunochemistry has improved the analytical sensitivity of these methods, reduced their cost and universalized some of the reagents used [99,103]. These methods are currently widely used to construct immuno- and histochemical tests used in in vitro diagnostics to determine the concentration of various hormones, proteins, antigens, tumor markers, immunoglobulins (including tIgE and sIgE) and many different analytes capable of forming immunological complexes with their specific antibodies. Biotin supplementation is a significant factor interfering with immunochemical methods using biotin–avidin or biotin–streptavidin binding by blocking or excessive capture of avidin or streptavidin active reagents. This can lead to false negative results (e.g., by blocking detection reagents—labeled streptavidin) or false positive results (e.g., excessive binding of detection reagents to the test matrix) [9,104,105,106,107,108,109,110,111,112,113]. The analytical strategy based on the use of biotin–streptavidin binding is also used in some analytical systems for total and specific IgE assays, such as 3gAllergy IMMULITE-2000 (Siemens Healthcare Diagnostics, Tarrytown, NY, USA) and NOVEOS (HYCOR Biomedical, Garden Grove, CA, USA), which may have an impact on the results of IgE assays obtained using these analytical platforms in individuals supplementing with high doses of vitamin B7. Biotin interference in the analytical procedure is independent of the target allergen [114,115]. Biotin supplementation, regardless of the dose taken, does not affect the results of total IgE and specific IgE determinations performed on ImmunoCAP analyzers (Thermo Fisher Scientific, Phadia AB, Uppsala, Sweden), because the analytical methodology of this system does not use a biotin–streptavidin bridge in the strategy of this analytical procedure [114].

The best way to avoid biotin interference in immunochemical reactions using biotin–streptavidin bridges is to stop the patient from supplementing with vitamin B7 at least 48 h before blood is drawn for laboratory tests [9,116]. More detailed time frames for stopping oral vitamin B7 are also available. Depending on the dose and body weight used, it is recommended to stop biotin supplementation 8 h before sampling in patients taking biotin at doses of 10 mg/day, for 3 days in the case of doses of 100–300 mg/day, and for 7 days in the case of children taking 2 and 15 mg/kg/day [117]. In any case, it is recommended that the laboratory performing the analysis be informed if the patient is taking vitamin B7 [22].

It is also very important to educate medical personnel involved in the diagnostic process and patient care (doctors, laboratory specialists, nurses, dietitians) about possible interference in analytical methods. This is also important because these personnel, especially clinicians, nurses and dietitians, have direct contact with patients, which translates into a significant participation of these professionals in educating patients on how to properly prepare for laboratory tests. This allows many errors to be avoided, improves the quality and effectiveness of diagnostics, shortens its duration and reduces costs [9,10,109,118,119,120].

## 5. Biotin Hypersensitivity—Diagnostic Possibilities

Diagnostics of biotin hypersensitivity can be difficult due to the lack of standardized tests for both in vivo and in vitro diagnostics. As can be seen in the available clinical descriptions of cases in which hypersensitivity to vitamin B7 or its precursors was suspected, the diagnostic process primarily used epidermal patch tests with the native substance indicated by the patient as a potential cause of the observed reactions. The decisive basis for diagnostics was a detailed clinical interview, medical history and analysis of the patient’s symptoms and the circumstances in which they occurred [43,44]. It seems, therefore, that in the diagnosis of biotin hypersensitivity, the focus should be primarily on a detailed analysis of the factors reported by the patient, and the participation of non-obvious factors should always be taken into account in the diagnostic process, including supplementation with commonly available vitamin preparations, used cosmetics and occupational exposure to various environmental substances that are potentially non-allergens.

## 6. Summary

Biotin (vitamin B7) is a naturally occurring water-soluble vitamin from the B group. Although only plants and some microorganisms have the ability to synthesize biotin, the ability to store it in animal tissues means that this vitamin is commonly found in many food products of both plant and animal origin. Biotin is also a common ingredient in pharmaceuticals, dietary supplements, foods and cosmetics. Despite the common use of biotin, even in high doses, it does not seem that hypersensitivity to vitamin B7 is a significant problem. So far, only two cases of hypersensitivity reactions to biotin or precursor substances for this vitamin have been published.

However, due to the significant role of biotin in modulating the function of the immune system, it seems very important to maintain the correct level of this vitamin in the body. As it results from the available data and taking into account the wide range of metabolic processes in which biotin is involved, both deficiency and excess of vitamin B7 in the body may have an adverse effect on immunological efficiency. Disturbance of the homeostasis of the immune system and dysfunction of the skin at the cellular level may result in an inflammatory response and hypersensitivity to various allergens or haptens.

The small amount of available data does not allow for unequivocal conclusions in this area. Hypersensitivity to biotin and the participation of vitamin B7 in modulating the function of the immune system, including hypersensitivity reactions, certainly require further research.

Another important aspect related to biotin supplementation is the fact that this vitamin may interfere with some analytical procedures that use strategies based on biotin-streptavidin (avidin) binding. This may affect the results of some laboratory tests, including total IgE and specific IgE. Therefore, it is necessary to take into account whether the patient is taking preparations containing biotin and to recommend that they stop supplementing with this vitamin at least 48 h before blood collection for testing or choose a method free from vitamin B7 interference. It is also necessary to educate medical personnel directly involved in preparing the patient for the test and educate them in this area. Such a procedure will significantly contribute to shortening the time to diagnosis, improving the quality, and reducing the costs of diagnostics.

## Figures and Tables

**Table 1:** Natural sources of vitamin B7 [38].

Food Product	Biotin Content [ng/g]
Eggs, milk, dairy products	
Hens egg whole (cooked) *	214
Hens egg white (cooked) *	58
Hens egg yolk (cooked) *	272
Cow milk (2% fat)	1.13
Cheddar cheese	14
Meat and fish	
Chicken liver (fried)	1872
Beef liver (fried)	416
Turkey ham	7.3
Salmon (water-cured)	59
Tuna (water-cured)	6.82
Fruits and vegetables (fresh and processed)	
Strawberries (fresh)	15
Avocados (fresh)	9.61
Raisins	3.91
Raspberries (fresh)	1.78
Bananas (fresh)	1.33
Orange (fresh)	0.49
Orange juice (reconstituted from concentrate)	4.13
Apple (fresh)	0.2
Apple juice (reconstituted from concentrate)	0.52
Sweet potatoes (cooked)	14.5
Broccoli (fresh)	9.43
Spinach (frozen)	7.05
Tomatoes (fresh)	7.01
Carrots (canned)	6.22
Cauliflower (fresh)	1.61
Mushrooms and yeast	
Mushrooms (canned)	21.6
Yeast	202
Nuts and seeds	
Peanuts (roasted, salted)	175
Pecans (fresh)	20
Almonds (roasted, salted)	44.07
Walnuts (fresh)	25.9
Sunflower seeds (roasted, salted)	78
Cereals and bread	
Oat flakes	1.91
Whole grain bread	0.74

* Biotin content in eggs is given for cooked eggs. Biotin bioavailability from raw eggs (especially egg whites) is low because raw egg whites contain avidin, which binds biotin and blocks its absorption [35].

## Data Availability

Not applicable.
